# Current Practice of Imaging-Guided Interventional Procedures in Rheumatic and Musculoskeletal Diseases: Results of a Multinational Multidisciplinary Survey

**DOI:** 10.3389/fmed.2021.779975

**Published:** 2021-11-22

**Authors:** Francesco Carubbi, Philipp Bosch, Pedro M. Machado, Carlo Alberto Scirè, Alessia Alunno, Fabian Proft, Xenofon Baraliakos, Christian Dejaco

**Affiliations:** ^1^Internal Medicine and Nephrology Unit, Department of Life, Health and Environmental Sciences, University of L'Aquila, L'Aquila, Italy; ^2^Department of Medicine, ASL 1 Avezzano-Sulmona-L'Aquila, San Salvatore Hospital, L'Aquila, Italy; ^3^Division of Rheumatology and Immunology, Department of Internal Medicine, Medical University of Graz, Graz, Austria; ^4^Department of Neuromuscular Diseases, Centre for Rheumatology University College London, London, United Kingdom; ^5^National Institute for Health Research (NIHR) University College London Hospitals Biomedical Research Centre, University College London Hospitals NHS Foundation Trust, London, United Kingdom; ^6^Department of Rheumatology, Northwick Park Hospital, London North West University Healthcare NHS Trust, London, United Kingdom; ^7^Rheumatology Unit, Department of Medical Sciences, University of Ferrara, Ferrara, Italy; ^8^Epidemiology Research Unit, Italian Society for Rheumatology, Milan, Italy; ^9^Department of Gastroenterology, Infectiology and Rheumatology (Including Nutrition Medicine), Charité—Universitätsmedizin Berlin, Corporate Member of Freie Universität Berlin and Humboldt-Universität zu Berlin, Berlin, Germany; ^10^Rheumazentrum Ruhrgebiet Herne, Ruhr-University, Bochum, Germany; ^11^Department of Rheumatology, Hospital of Bruneck (ASAA-SABES), Bruneck, Italy

**Keywords:** interventional procedures, imaging, ultrasonography, rheumatic and musculoskeletal diseases, survey

## Abstract

**Objectives:** To investigate opinion and routine practice of specialists from different disciplines on imaging techniques for interventional procedures related to rheumatic and musculoskeletal diseases (RMDs).

**Methods:** An English-language questionnaire was developed by an international working group and distributed to health care providers of various disciplines involved in the care of people with RMDs via an online survey tool (SoSci Survey®) from December 2019 to May 2020.

**Results:** A total of 1,105 respondents from 56 countries completed the survey, over 60% of participants were rheumatologists. The majority of respondents (88%) performed interventional procedures in RMDs patients and 90% of them used imaging guidance. Ultrasonography was the most frequently used technique, particularly among rheumatologists. X-ray and computed tomography were mainly used by radiologists. A discrepancy emerged between the importance assigned to certain items such as the availability of a second operator and their actual implementation in clinical practice. Local barriers, lack of resources and facilities were mentioned as the most relevant obstacles in this regard. Lack of training on imaging and/or imaging guided procedures did not emerge as a barrier to perform such interventions; in fact, 19% of respondents performing the procedures indicated not to have received adequate training in this field.

**Conclusions:** This is the first multinational multidisciplinary survey exploring in detail the opinions and practice on imaging guidance for interventional procedures in RMDs. A harmonization of protocols based on international guidelines, along with adequate training programmes and interventions on barriers at national/local levels are the main unmet needs requiring attention.

## Introduction

Over the last decades, imaging has been increasingly used in the diagnostic and therapeutic workup of patients with suspected or established rheumatic and musculoskeletal diseases (RMDs) (1, 2). Historically, conventional radiology was the most widespread technique in the field; however, the advent of other methodologies enabling an earlier detection of abnormalities and a more detailed assessment of anatomical structures prompted their implementation in clinical practice (3–10). Musculoskeletal ultrasonography (MSUS), for example, is commonly used by rheumatologists and radiologists, albeit to a different extent across countries (11, 12). In contrast, fluoroscopy, radiography, magnetic resonance imaging (MRI) and computed tomography (CT) are predominantly performed by radiologists (13).

In recent years, imaging has increasingly been adopted to guide interventional procedures related to RMDs (2, 14, 15). A survey conducted in 2012 reported that in almost every European country, US-guided aspiration or injection was performed by a small proportion (<10%) of rheumatologists routinely (16). Another survey from 2019 found that in the majority of European Alliance of Associations for Rheumatology (EULAR) countries, <50% of rheumatology regularly used MSUS (13). In the same survey, it was reported that <20% of rheumatologists conducted US guided injections of joints and other musculoskeletal structures (13).

Recently, the European Society of Musculoskeletal Radiology (ESSR) published a series of consensus papers agreeing upon a number of clinical indications for image-guided interventional procedures in the musculoskeletal system (17). A multidisciplinary approach to this topic, taking into account the possible barriers to implement standardized protocols outside radiology, however, is lacking. Besides, we do not know to what extend ultrasound is used for other interventional procedures in patients with RMDs and to what extend fluoroscopy, MRI and other imaging techniques are applied by rheumatologists, radiologists and other professionals in clinical practice (13).

Because of these gaps in evidence, we conducted a multinational, multidisciplinary survey with the following objectives: (1) to investigate whether and how frequently imaging techniques are used for interventional procedures by different specialists involved in the care of people with RMDs; (2) to explore the technical standards applied to imaging guided interventions employed in different settings; and (3) to understand the perception of specialists about the importance of technical standards for imaging guided procedures.

The results of this survey, along with a systematic literature review, informed the EULAR Points to Consider for the use of imaging to guide interventional procedures in patients with RMDs (18, 19).

## Materials and Methods

### Survey Structure

An English-language questionnaire was developed by an international working group composed of rheumatologists with long-standing experience in imaging and imaging guided procedures and a methodologist. This on-line based questionnaire contained 29 questions organized in two sections. The majority of questions were in the multiple-choice format, recognizing the possibility that multiple not mutually exclusive strategies might have been applied. The survey also contained a few single choice (e.g., for age and sex) or open-ended questions. Section Introduction included questions on demographics, current practice of interventional procedures in patients with RMDs and the use of imaging guidance. According to the answers given (performing/not performing interventional procedures; utilizing/not utilizing imaging guidance), respondents were redirected to different parts of section Materials and Methods. Respondents declaring not to perform interventional procedures were questioned to express their opinion on the importance of specific settings, such as using a sterile operating room or applying a sterile cover to ultrasound probes. Conversely, those who performed interventional procedures in clinical routine were asked to provide additional details about their practice along with their opinion about the importance of the settings mentioned above. See Supplementary File 1 for the full questionnaire.

To ensure a broad distribution of the survey and to obtain a representative sample, we sought the support of European and American national scientific societies of rheumatology, radiology, orthopedics, pediatrics, sports medicine, physical medicine and rehabilitation, neurology, neurosurgery, anaesthesiology and family medicine. Young investigators groups such as the Emerging EULAR Network (EMEUNET) and the Young Club of the European Society of Musculoskeletal Radiology (ESSR) were also involved. The working group members also personally contacted physicians and other health care professionals in rheumatology (HPR) from different countries, requesting them to answer and disseminate the questionnaire (snow-ball principle).

The survey was conducted via an online survey tool (SoSci Survey®) from December 2019 to May 2020. It was accompanied by an explanatory letter regarding the purpose of the study and a request of agreement to use the data for statistical analyses. The target population was health care providers of various disciplines involved in the care of people with RMDs.

Ethical approval was not required because the study did not involve patients; all responses were anonymous. Patients were not involved in the design of the study.

### Statistical Analysis

Data were imported from the survey platform into IBM SPSS 23.0. Descriptive statistics were used to summarize the data. Absolute and relative frequencies were calculated and depicted in tabular and graphical form. Data are presented as number (nominator) and percentage of all available responses to each question (denominator) throughout the manuscript. The denominator may change from question to question for the following reasons: (1) questions and individual answers could have been skipped, (2) some questions could have been answered with “not applicable” or “do not know,” which were subtracted from the denominator as indicated, (3) specific subgroup analyses were conducted. Since the majority of questions were in the multiple-choice format, the sum of nominators from individual questions may exceed the corresponding denominator. Comparisons between groups were performed with the Mann-Whitney *U*-test and *p* < 0.05 were considered statistically significant.

## Results

### Demographic Characteristics of Respondents as Well as Current Practice of Imaging and Interventional Procedures in Patients With RMDs

A total of 1,105 respondents from 56 countries completed the survey (see Table 1 for demographics). Over 60% of participants were rheumatologists, 11% were radiologists, 10% specialists in physical medicine and rehabilitation while the remainder was a heterogeneous group of physicians and HPR. About half of respondents worked in a university hospital. There was a wide country representation, with the 3 top countries being Italy (19% of responses), Turkey (13%) and Germany (11%). Nine hundred and seventy-eight (88%) respondents reported to perform interventional procedures in patients with RMDs, 90% of them (*n* = 885) under imaging guidance.

**Table 1:** Characteristics of survey respondents (*N* = 1,105).

**Variable**	**N (%)**
**Gender**	
M	470 (43)
F	635 (57)
**Age**	
≤ 30	106 (10)
31–35	203 (18)
36–39	189 (17)
40–49	282 (26)
≥50	325 (29)
**Specialty**	
Rheumatology	705 (64)
Radiology	120 (11)
Physical medicine and rehabilitation	115 (10)
Anesthesiology/pain medicine	55 (5)
Orthopedics	42 (4)
Health professional in rheumatology	32 (3)
Pediatrics	25 (2)
Sports medicine	20 (2)
General practice	19 (2)
Neurology	11 (1)
Other	32 (3)
**Institution**	
University hospital	542 (50)
Hospital	359 (33)
Private practice	172 (14)
Other	32 (3)
**Country**	
Italy	212 (19)
Turkey	148 (13)
Germany	124 (11)
Switzerland	82 (7)
Austria	67 (6)
Netherlands	64 (6)
Portugal	63 (6)
Denmark	51 (5)
Spain	39 (3)
France	37 (3)
Other	218 (20)

The most frequent procedures were aspiration/injection of large joints (95%), tendon/tendon sheet/enthesis/bursae (80%) and small joints (79%). Soft tissue injections were conducted by 59% of respondents while 27 and 26% reported to perform spine injection and nerve blockade, respectively. About 10% of respondents (also) conducted other procedures, with salivary gland and abdominal fat biopsies being the most frequent ones (Table 2). No significant association was found between specific interventional procedures and age, specialty or country.

**Table 2:** Number (percentage) of respondents reporting to perform one or more of the indicated procedures.

**Procedure**	**N (%)**
Joint aspiration/injection (large joints)	925 (95)
Tendon/tendon sheet/enthesis/bursae aspiration/injection	777 (80)
Joint aspiration/injection (small joints)	770 (79)
Soft tissue injection	576 (59)
Spine injection	267 (27)
Nerve blockade	253 (26)
Synovial biopsy	136 (14)
Muscle biopsy	118 (12)
Nerve biopsy	27 (3)
Other	82 (8)

**Figure 1:**
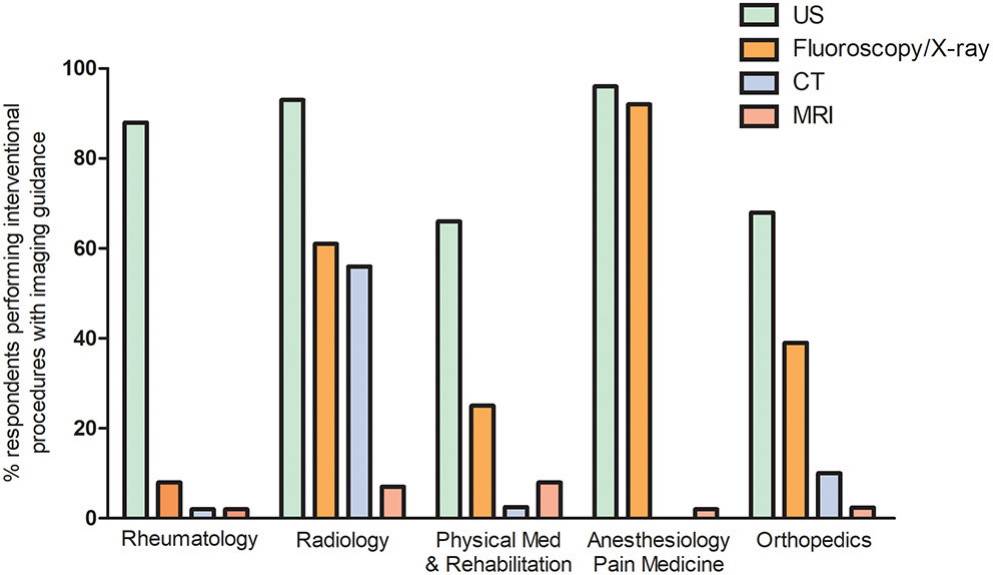
Percentage of respondents using ultrasonography, X-ray/fluoroscopy, computed tomography or magnetic resonance imaging to guide interventional procedures, divided by specialty. This question was answered only by respondents stating that they performed imaging guided procedures. The 5 specialties with the highest numbers of respondents are depicted.

The 885 participants who answered that they used imaging guidance to conduct interventional procedures were asked to provide additional details about their current practice. As shown in Table 3, over 90% of respondents reported to use imaging guidance for injection/aspiration of large joints while a lower proportion conducted imaging guided injection/aspiration of small joints (78%) or tendons/tendon sheets/entheses/bursae (73%). Fifty-seven percent of respondents stated that they performed the entire procedure under direct imaging guidance, mostly using ultrasonography (US) (97%), followed by fluoroscopy/X-ray (27%). The imaging techniques applied varied across specialties (Figure 1): US guidance was conducted by the majority of radiologists, whereas anaesthesiologists/pain doctors and orthopedic surgeons also largely used fluoroscopy/X-ray. More than half of radiologists also performed CT guided interventions. CT was rarely used by other specialists and MRI was rarely applied for this purpose by any group.

**Table 3:** Number (percentage) of respondents reporting to apply any of the imaging modalities or techniques for imaging guided interventions as indicated.

**Interventional procedures supported by imaging guidance**	**N (%)**
Joint aspiration/injection in large joints	808 (92)
Joint aspiration/injection in small joints	695 (78)
Tendon/tendon sheet/enthesis/bursae aspiration/injection	650 (73)
Soft tissue injection	297 (34)
Spine injection	242 (27)
Nerve blockade	205 (23)
Synovial biopsy	138 (16)
Muscle biopsy	101 (11)
Nerve biopsy	23 (3)
Other	41 (5)
**Approach toward imaging guided interventional procedures**	
Direct imaging guidance for the whole procedure	504 (57)
Use of imaging to find the appropriate anatomical landmark followed by blind performance of the procedure (indirect method)	186 (21)
Use of both direct and indirect methods of imaging guidance depending on the anatomical location	195 (22)
**Imaging technique used to guide interventional procedures**
US	860 (97)
Fluoroscopy/X-ray	237 (27)
CT	100 (11)
MRI	35 (4)
Other imaging technique	6 (1)
**Application of contrast agent to control correct position of the needle**	
Never	628 (71)
Sometimes	195 (22)
Always	62 (7)
**Use of air to control correct position of the needle**	
Never	681 (77)
Sometimes	177 (20)
Always	27 (3)

Among respondents performing interventional procedures using imaging guidance, 57% conducted the entire interventional procedure under direct imaging guidance, 22% used imaging to find the appropriate anatomical landmark and then performed the procedure blindly (indirect method) and 21% used both techniques depending on the situation. In the latter group, site, type of procedure, anatomical complexity of the area, amount of fluid in the joint, assistance from a colleague or HPR, patient collaboration and time available were the factors influencing the choice of either the direct of the indirect injection technique. A contrast agent or air were rarely applied to control for needle placement.

**Figure 2:**
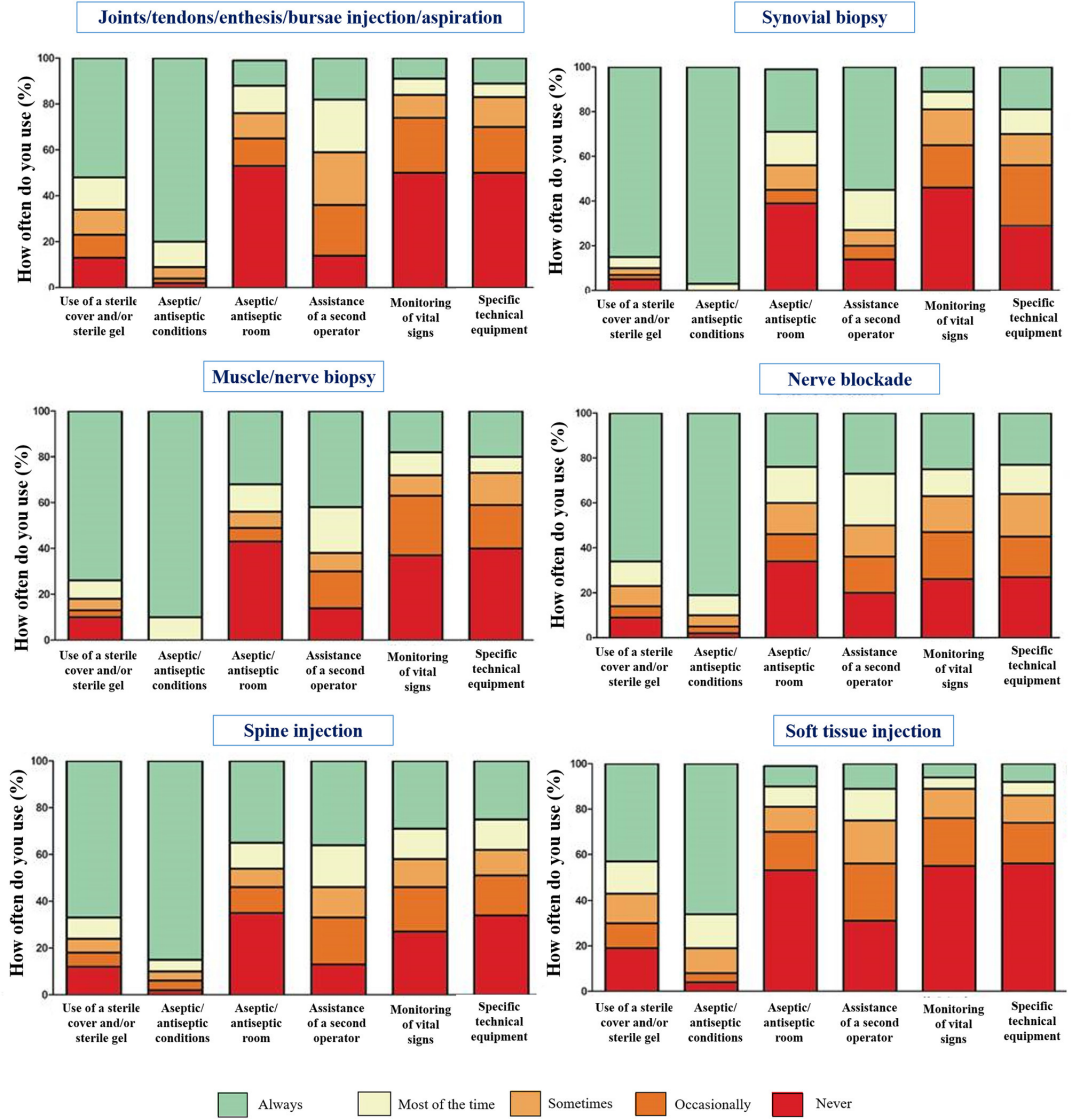
Frequency of use of different preparations and procedural steps in respondents performing interventional procedures according to site and type of interventions.

Next, respondents were asked to indicate what settings and protocols they used for different imaging guided interventions in clinical practice. As depicted in Figure 2, sterile covers for the ultrasound probe, a sterile room (level of sterility not further specified), and assistance of a second operator were commonly considered for complex interventions such as injection of the spine or synovial biopsy. In contrast, soft tissue or joint/tendon/entheses injections were mostly conducted without assistance and with less effort to keep the setting aseptic. Stratifying responses by specialty revealed no significant differences between groups.

Ninety-three participants did not use imaging guidance to perform interventional procedures in patients with RMD for the following reasons: lack of facilities (53%), preference to send patients to another specialist (mainly a radiologist) who performed the procedures under imaging guidance (26%), opinion that imaging guidance did not provide any benefit over clinical guidance (24%), lack of training (10%), insurance/legal issues (2%).

### Perception of the Importance of Individual Preparations and Procedural Steps to Conduct Imaging Guided Interventional Procedures

**Figure 3:**
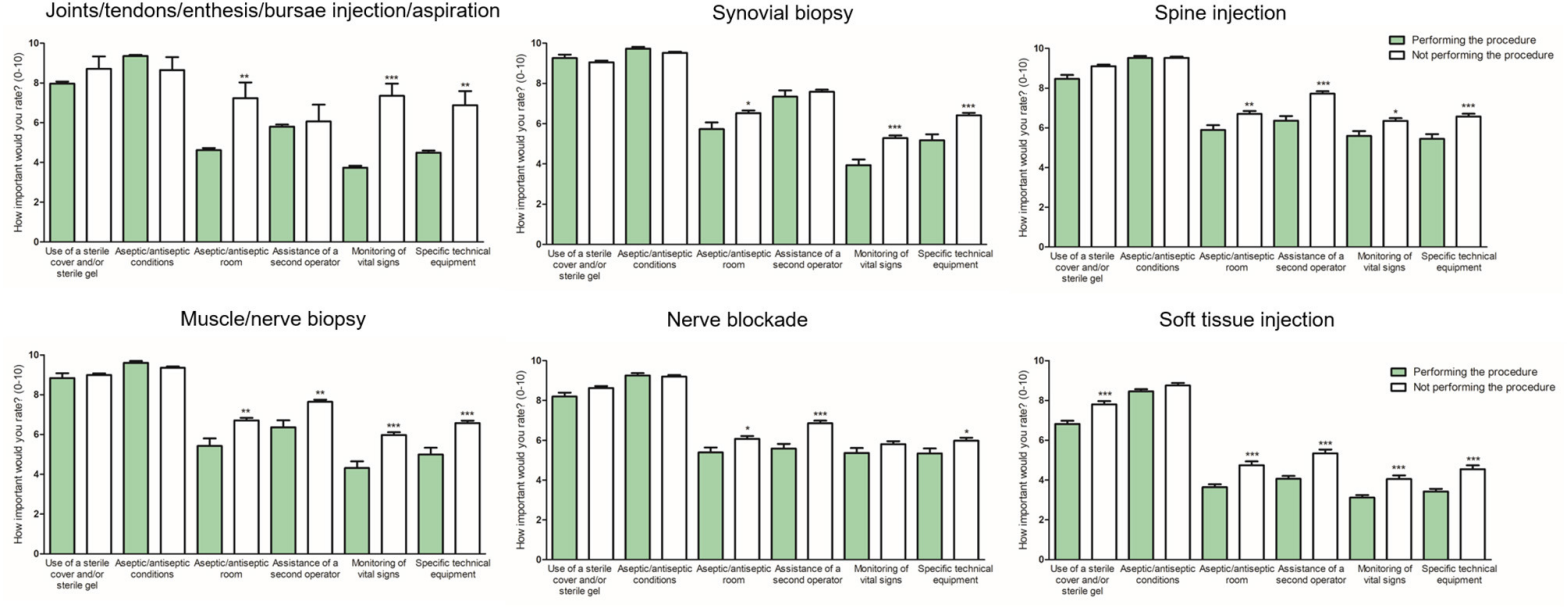
Importance of specific preparations and settings according to respondents performing/not performing interventional procedures. Bars indicate mean and standard error of the mean. ***p < 0.001, **p < 0.01, and *p < 0.05.

All participants, regardless of whether they performed imaging-guided procedures or not, were asked to rate the importance of a number of preparations and procedural steps on a scale ranging from 0 (least importance) to 10 (highest importance). Respondents who used imaging to guide interventions in clinical practice were also asked whether there was any discordance between the perceived importance of any given preparation/step and its actual implementation in clinical practice. Figure 3 compares responses from those who actually use imaging guidance with those who do not use this technique to guide interventions. Overall, application of a sterile cover and/or sterile gel as well as aseptic conditions received the highest ratings by all respondents. Furthermore, respondents who did not perform imaging guided procedures rated some items higher than those who regularly used imaging, such as the relevance of an aseptic room, monitoring vital signs, application of specific technical equipment or the assistance of a second operator. Based on free-text comments of some respondents it seems that insufficient resources (e.g., unavailability of a sterile room or a second operator) was the main explanation for the discordancy between the perceived importance and actual lack of implementation of these factors in clinical practice. When analyzing the data according to specialty, some similarities and differences emerged between rheumatologists and radiologists (Supplementary Table 1). Overall, there was an agreement between the 2 groups, however radiologists rated some items for spine injections, nerve/muscle biopsy and nerve blockade lower than rheumatologists (all *p* < 0.05).

### Training on Imaging and/or on Imaging Guided Interventional Procedures

Respondents performing interventional procedures were asked to provide details about their training in this field. Nineteen percent of respondents reported to have been trained in imaging, 8% in imaging guided procedures and 54% in both; however, 19% of respondents did not receive any specific training. When stratified by specialty, 15.2% of rheumatologists and 5.4% of radiologists stated to have never been trained specifically (*p* = 0.03). The majority of participants received training in US (73%), 25% in fluoroscopy/X-ray, 10% in CT, 5% in MRI and 1% in other imaging techniques. While rheumatologists where almost exclusively trained in US, radiologists were trained in US, X-ray, CT and to a lesser extent in MRI (Supplementary Figure 1). When analyzing data by country, we observed that none of the respondents from some Eastern and Northern European Countries (e.g., Norway, Poland, Serbia) reported lack of training. Conversely, for some Countries such as Italy and Denmark although the majority of respondents reported having been trained, some of them reported not having been trained. These data, however, has to be interpreted with caution because of the low response rate in several countries.

Heterogeneity was observed with regard to the level of training. Several participants, mainly rheumatologists, reported to have attended one or more EULAR US courses and/or courses organized by National Societies for rheumatology/ultrasonography. Others stated to have been taught by local senior residents/attending physicians or during fellowships at other institutions but not to have attended courses. Cadaver training and courses not otherwise detailed were mentioned by a subgroup of respondents only.

## Discussion

This is the first survey exploring in detail the implementation and technical standards of imaging guidance for interventional procedures by different specialists involved in the care of people with RMDs across several countries. We observed that a large number of rheumatologists and specialists from other disciplines perform interventional procedures, and the majority of them use imaging guidance. However, we cannot rule out that people who have no interest or skill in this filed were less motivated to complete the survey thus introducing some bias into our results. Furthermore, although we addressed European and American scientific societies alongside individual experts from several specialities in addition to rheumatology, the proportion of specialists other than rheumatologists completing the survey was relatively low and respondents were predominately from western European countries. This hampered the possibility to map in detail the implementation of specific protocols according to the geography and specialty. Our data are therefore not meant to accurately reflect the clinical implementation of imaging and imaging guidance of interventions worldwide, but rather to explore which imaging techniques as well as what protocols and settings are commonly used in daily routine.

Mandl et al. already observed in 2019 a higher uptake of MSUS by rheumatologists, both for diagnosis and monitoring of RMDs, as compared to previous surveys from 2010 and 2012 (12, 13, 16). In 2010, it was estimated that in 56% of EULAR countries <10% of rheumatologists performed MSUS routinely (12). According to a survey from 2012, US-guided aspiration or injection was performed only by a very small proportion (<10%) of rheumatologists routinely (16). More recently, another survey found that the percentage of rheumatologists performing US was highly variable, ranging from more than 80% in 6% of EULAR countries to <10% in 15% of EULAR countries (13). In the same survey, it was observed that in the majority of EULAR countries, <20% of rheumatologists use US to guide injections of joints and other musculoskeletal structures (13).

In our survey, 90% of respondents reported that they performed imaging-guided interventional procedures, and that US was the most frequent imaging technique used, particularly among rheumatologists. This supports the perception of the increasing importance and widespread use of this methodology.

We further observed that X-ray and CT are still mainly performed by radiologists even though a high proportion of orthopedics and anesthetists utilized X-ray to guide interventional procedures.

US is most frequently applied for injections of joints, tendons, tendon sheaths and bursae. The decision to perform either the whole procedure under direct imaging guidance or to use imaging to find the appropriate anatomical landmark and then to conduct the procedure blindly was related to a number of factors such as the anatomical area, time constraints and/or the lack of a second operator. The discrepancy between the importance respondents assigned to certain preparations such as the availability of a second operator or monitoring vital signs, as compared to the actual implementation of these procedures in clinical practice in noteworthy. Local barriers, lack of resources and facilities were mentioned as the most relevant obstacles across countries.

Lack of training on imaging and/or imaging guided procedures did not emerge as a barrier to perform such interventions given that 19% of respondents who regularly conduct imaging guided procedures indicated not to have received adequate training in this field. While this might be worrisome to some extent, it also indicates the need for specific training curricula and dedicated courses. Although several national and international societies (e.g., EULAR) have organized courses in MSUS and other imaging techniques, the offers for imaging guided interventions as well as training possibilities on fluoroscopy/X-ray, MRI, CT or positron emission tomography are still limited (13). In some countries, competencies in MSUS, but not in imaging guided interventions, have yet to be included in training curricula for rheumatologists.

Our study is limited by the descriptive nature and by a potential responder bias. For example, there were more responses from Switzerland and the Netherlands, countries with a relatively small population, than from France and Spain. We followed the same dissemination strategy of the survey in every country, so any imbalance in the number of responses compared to the expected target population may be due to factors beyond our control (e.g., different communication strategies of national societies). In addition, the respondents of this survey might be biased by enthusiasm for imaging and imaging guided procedures and are thus only to a limited extent representative for current clinical practice in Europe and other countries (13, 16). Furthermore, more than 60% of the respondents were rheumatologist hence our results might not reflect the global medical opinion about guided procedures. Nevertheless, our data help to stimulate research, highlight the training requirements of rheumatologists and other specialists and may ultimately be useful to organizers of imaging courses and national societies/authorities deciding on national training curricula. The main strength of our study is the large sample size including rheumatologists, radiologists and other specialists from several countries, thereby providing a comprehensive picture of the implementation and technical standards of imaging guidance for interventional procedures in patients with RMDs. It highlights the need for further research in order to be able to define standardized protocols for specific interventions which should ultimately increase the quality of care.

In conclusion, our study demonstrates that rheumatologists and other health care providers across Europe who have an interest in the field of imaging, perform US and other imaging-guided interventions. However, the protocols are heterogeneous and influenced by several factors, in particular the anatomical site and the type of intervention performed. Such heterogeneity hampers the comparison of real-life data from different settings and underscores the need to define standardized protocols based on international guidelines. Furthermore, we observed that there are several barriers to optimize the procedures in clinical practice and that adequate training programs are needed.

## Data Availability Statement

The original contributions presented in the study are included in the article/Supplementary Material, further inquiries can be directed to the corresponding author/s.

## Ethics Statement

Ethical review and approval was not required for the study on human participants in accordance with the local legislation and institutional requirements. Written informed consent for participation was not required for this study in accordance with the national legislation and the institutional requirements.

## Author Contributions

FC and PB drafted the first version of the manuscript. All authors contributed to the study design, interpretation of data, revised critically the manuscript, and approved the final version.

## Funding

PMM was supported by the National Institute for Health Research (NIHR), University College London Hospitals (UCLH), Biomedical Research Centre (BRC).

## Conflict of Interest

FC has received consulting/speaker's fees from Abbvie and Celgene, all unrelated to this manuscript. PMM has received consulting/speaker's fees from Abbvie, BMS, Celgene, Eli Lilly, Janssen, MSD, Novartis, Orphazyme, Pfizer, Roche and UCB, all unrelated to this manuscript. FP has received research grants from Novartis, Lilly and UCB and received consulting/speaker's fees from Abbvie, AMGEN, BMS, Hexal, Janssen, MSD, Novartis, Pfizer, Roche and UCB, all unrelated to this manuscript. XB has received consulting/speaker's fees from Abbvie, BMS, Celgene, Galapagos, Gilead, Eli Lilly, Janssen, MSD, Novartis, Pfizer and UCB, all unrelated to this manuscript. CD has received consulting/speaker's fees from Abbvie, Eli Lilly, Janssen, Novartis, Pfizer, Roche and Sanofi, all unrelated to this manuscript. The remaining authors declare that the research was conducted in the absence of any commercial or financial relationships that could be construed as a potential conflict of interest.

## Publisher's Note

All claims expressed in this article are solely those of the authors and do not necessarily represent those of their affiliated organizations, or those of the publisher, the editors and the reviewers. Any product that may be evaluated in this article, or claim that may be made by its manufacturer, is not guaranteed or endorsed by the publisher.
