# Supplementary material for: Current Practice of Imaging-Guided Interventional Procedures in Rheumatic and Musculoskeletal Diseases: Results of a Multinational Multidisciplinary Survey
Source: Front Med (Lausanne). 2021 Nov 22;8:779975. doi: 10.3389/fmed.2021.779975 (PMC8645558; doi:10.3389/fmed.2021.779975)
Supplement: Supplementary file 1 [file Data_Sheet_1.docx]

**Online supplementary text S1**

**Structure of the survey**

1. What is your age?

≤ 30

31 –35

36 – 39

40 - 49

≥ 50

2. What is your gender?

Female

Male

3. What is your current position? (Several answers possible)

Rheumatologist in training

Rheumatologist (post-training)

Radiologists in training

Radiologists (post training)

Orthopaedics in training

Orthopaedics (post training)

Sports medicine doctors in training

Sports medicine doctors (post training)

Physical medicine and rehabilitation fellow in training

Physical medicine and rehabilitation doctor post training

Neurologist in training

Neurologist (post training)

Paediatrician in training

Paediatrician (post training)

General Practitioner/Family Medicine doctor in training

General Practitioner/Family Medicine doctor (post training)

Non-clinical researcher

Health professional in Rheumatology

Other [please specify]:

4. Country of work [Drop down list of countries]

5. Type of institution

University Hospital

Hospital

Private practice

Other [please specify]

6. Do you perform interventional procedures related to musculoskeletal diseases (e.g. joint aspiration/injection, spine injection, muscle biopsy)

Yes

No

*Note: if the answer is no, the participant is redirected to Q15 and survey ends after Q21*

7. If yes, which procedures do you perform? (Several answers possible)

Joint aspiration/injection in large joints: (e.g. shoulders, elbows, hips, knees, ankles)

Joint aspiration/injection in small joints (e.g. wrists, fingers/toes)

Tendon/tendon sheet/enthesis/bursae aspiration/injection

Spine injection

Synovial biopsy

Muscle biopsy

Nerve biopsy

Nerve blockade

Soft tissue injection

Other [please specify]

8. Do you use **imaging guidance** to perform interventional procedures related to musculoskeletal diseases?

Yes

No

9. If no, why? (Several answers possible)

Lack of facilities (e.g. no ultrasound machine at my center, lack of assistance of a second operator, lack of an environment with aseptic/antiseptic conditions)

Insurance/legal issues

I send the patient to another specialist/health professional that performs these procedures with imaging guidance [please specify what kind of specialist]

I only perform blind procedures because I think that imaging guidance does not provide any benefit compared to blind procedures

Other [please specify]

*Note: if the answer is no, the participant is redirected to Q15 and survey ends after Q28*

10. If yes, for which procedure do you use imaging guidance? (Several answers possible)

Joint aspiration/injection in big joints: (e.g shoulders, elbows, hips, knees, ankles)

Joint aspiration/injection in small joints (e.g. wrists, fingers/toes)

Tendon/tendon sheet/enthesis/bursae aspiration/injection

Spine injection

Synovial biopsy

Muscle biopsy

Nerve biopsy

Nerve blockade

Soft tissue injection

Other [please specify]

11. How do you use imaging guidance?

I perform the whole procedure using direct imaging guidance

I use imaging to find the appropriate anatomical landmark and then I perform the procedure blindly

I use both methods depending on the situation [please specify]

12. Which imaging technique do you use? (Several answers possible)

US

Fluoroscopy/X-ray

CT

MRI

other imaging technique [please specify]

13. Do you use a contrast agent to control needle placement?

always

never

sometimes [please specify]

14. Do you use air to control needle placement?

always

never

sometimes [please specify]

15: From 0 (not important at all) to 10 (absolutely necessary), how important would you rate the following conditions/items for **joints/tendons/enthesis/bursae injection/aspiration**?

Use of a sterile cover and/or sterile gel (0-10)

Aseptic/antiseptic conditions (to maintain sterile conditions on tissues, on materials, and in rooms, e.g. an aseptic/antiseptic room, disinfection of probe, sterile gloves) (0-10)

Assistance of a second operator (0-10)

Monitoring of vital signs (0-10)

Specific technical equipment (e.g. needles with higher reflection, other specific devices…) (0-10)

16. From 0 (not important at all) to 10 (absolutely necessary), how important would you rate the following conditions/items for **synovial biopsy**?

Use of a sterile cover and/or sterile gel (0-10)

Aseptic/antiseptic conditions (to maintain sterile conditions on tissues, on materials, and in rooms, e.g. an aseptic/antiseptic room, disinfection of probe, sterile gloves) (0-10)

Assistance of a second operator (0-10)

Monitoring of vital signs (0-10)

Specific technical equipment (e.g. needles with higher reflection, other specific devices…) (0-10)

17. From 0 (not important at all) to 10 (absolutely necessary), how important would you rate the following conditions/items for **spine injections**?

Use of a sterile cover and/or sterile gel (0-10)

Aseptic/antiseptic conditions (to maintain sterile conditions on tissues, on materials, and in rooms, e.g. an aseptic/antiseptic room, disinfection of probe, sterile gloves) (0-10)

Assistance of a second operator (0-10)

Monitoring of vital signs (0-10)

Specific technical equipment (e.g. needles with higher reflection, other specific devices…) (0-10)

18. From 0 (not important at all) to 10 (absolutely necessary), how important would you rate the following conditions/items for **muscle/nerve biopsies**?

Use of a sterile cover and/or sterile gel (0-10)

Aseptic/antiseptic conditions (to maintain sterile conditions on tissues, on materials, and in rooms, e.g. an aseptic/antiseptic room, disinfection of probe, sterile gloves) (0-10)

Assistance of a second operator (0-10)

Monitoring of vital signs (0-10)

Specific technical equipment (e.g. needles with higher reflection, other specific devices…) (0-10)

19. From 0 (not important at all) to 10 (absolutely necessary), how important would you rate the following conditions/items for **nerve blockade**?

Use of a sterile cover and/or sterile gel (0-10)

Aseptic/antiseptic conditions (to maintain sterile conditions on tissues, on materials, and in rooms, e.g. an aseptic/antiseptic room, disinfection of probe, sterile gloves) (0-10)

Assistance of a second operator (0-10)

Monitoring of vital signs (0-10)

Specific technical equipment (e.g. needles with higher reflection, other specific devices…) (0-10)

20. From 0 (not important at all) to 10 (absolutely necessary), how important would you rate the following conditions/items for **soft tissue injection**?

Use of a sterile cover and/or sterile gel (0-10)

Aseptic/antiseptic conditions (to maintain sterile conditions on tissues, on materials, and in rooms, e.g. an aseptic/antiseptic room, disinfection of probe, sterile gloves) (0-10)

Assistance of a second operator (0-10)

Monitoring of vital signs (0-10)

Specific technical equipment (e.g. needles with higher reflection, other specific devices…) (0-10)

21. Did you receive specific training on imaging and/or on imaging-guided interventional procedures related to musculoskeletal diseases?

Yes, only on imaging [please specify the kind of training]

Yes, only on imaging-guided interventional procedures [please specify the kind of training]

Yes on both [please specify the kind of training]

No

22. On which imaging procedure used to guide interventional procedures related to musculoskeletal diseases did you receive training?

US

Fluoroscopy/X-ray

CT

MRI

Other [please specify]

23. How often do you use the following conditions/items for **joints/tendons/enthesis/bursae injection/aspiration**?

Use of a sterile cover and/or sterile gel (Never, occasionally, sometimes, most of the time, always, I do not perform this procedure)

Aseptic/antiseptic conditions (to maintain sterile conditions on tissues, on materials, and in rooms, e.g. an aseptic/antiseptic room, disinfection of probe, sterile gloves) (Never, occasionally, sometimes, most of the time, always, I do not perform this procedure)

Assistance of a second operator (Never, occasionally, sometimes, most of the time, always, I do not perform this procedure)

Monitoring of vital signs (Never, occasionally, sometimes, most of the time, always, I do not perform this procedure)

Specific technical equipment (e.g. needles with higher reflection, other specific devices…) ((Never, occasionally, sometimes, most of the time, always, I do not perform this procedure)

24. How often do you use the following conditions/items for **synovial biopsy**?

Use of a sterile cover and/or sterile gel (Never, occasionally, sometimes, most of the time, always, I do not perform this procedure)

Aseptic/antiseptic conditions (to maintain sterile conditions on tissues, on materials, and in rooms, e.g. an aseptic/antiseptic room, disinfection of probe, sterile gloves) (Never, occasionally, sometimes, most of the time, always, I do not perform this procedure)

Assistance of a second operator (Never, occasionally, sometimes, most of the time, always, I do not perform this procedure)

Monitoring of vital signs (Never, occasionally, sometimes, most of the time, always, I do not perform this procedure)

Specific technical equipment (e.g. needles with higher reflection, other specific devices…) (Never, occasionally, sometimes, most of the time, always, I do not perform this procedure)

25. How often do you use rate the following conditions/items for **spine injections**?

Use of a sterile cover and/or sterile gel (Never, occasionally, sometimes, most of the time, always, I do not perform this procedure)

Aseptic/antiseptic conditions (to maintain sterile conditions on tissues, on materials, and in rooms, e.g. an aseptic/antiseptic room, disinfection of probe, sterile gloves) (Never, occasionally, sometimes, most of the time, always, I do not perform this procedure)

Assistance of a second operator (Never, occasionally, sometimes, most of the time, always, I do not perform this procedure)

Monitoring of vital signs (Never, occasionally, sometimes, most of the time, always, I do not perform this procedure)

Specific technical equipment (e.g. needles with higher reflection, other specific devices…) (Never, occasionally, sometimes, most of the time, always, I do not perform this procedure)

26. How often do you use the following conditions/items for **muscle/nerve biopsies**?

Use of a sterile cover and/or sterile gel (Never, occasionally, sometimes, most of the time, always, I do not perform this procedure)

Aseptic/antiseptic conditions (to maintain sterile conditions on tissues, on materials, and in rooms, e.g. an aseptic/antiseptic room, disinfection of probe, sterile gloves) (Never, occasionally, sometimes, most of the time, always, I do not perform this procedure)

Assistance of a second operator (Never, occasionally, sometimes, most of the time, always, I do not perform this procedure)

Monitoring of vital signs (Never, occasionally, sometimes, most of the time, always, I do not perform this procedure)

Specific technical equipment (e.g. needles with higher reflection, other specific devices…) (Never, occasionally, sometimes, most of the time, always, I do not perform this procedure)

27. How often do you use the following conditions/items for **nerve blockade**?

Use of a sterile cover and/or sterile gel (Never, occasionally, sometimes, most of the time, always, I do not perform this procedure)

Aseptic/antiseptic conditions (to maintain sterile conditions on tissues, on materials, and in rooms, e.g. an aseptic/antiseptic room, disinfection of probe, sterile gloves) (Never, occasionally, sometimes, most of the time, always, I do not perform this procedure)

Assistance of a second operator (Never, occasionally, sometimes, most of the time, always, I do not perform this procedure)

Monitoring of vital signs (Never, occasionally, sometimes, most of the time, always, I do not perform this procedure)

Specific technical equipment (e.g. needles with higher reflection, other specific devices…) (Never, occasionally, sometimes, most of the time, always, I do not perform this procedure)

28. How often do you use the following conditions/items for **soft tissue injection**?

Use of a sterile cover and/or sterile gel (Never, occasionally, sometimes, most of the time, always, I do not perform this procedure)

Aseptic/antiseptic conditions (to maintain sterile conditions on tissues, on materials, and in rooms, e.g. an aseptic/antiseptic room, disinfection of probe, sterile gloves) (Never, occasionally, sometimes, most of the time, always, I do not perform this procedure)

Assistance of a second operator (Never, occasionally, sometimes, most of the time, always, I do not perform this procedure)

Monitoring of vital signs (Never, occasionally, sometimes, most of the time, always, I do not perform this procedure)

Specific technical equipment (e.g. needles with higher reflection, other specific devices…) (Never, occasionally, sometimes, most of the time, always, I do not perform this procedure)

29. Based on your answers is there a discordance between your impression of importance and the frequency of use of the various conditions/items?

Yes [please specify]

No

**Online supplementary Table S1**

**Importance of specific preparations and settings according to the respondent specialty.**

|  |  | **Rheumatologists** | | **Radiologists** | |  |
| --- | --- | --- | --- | --- | --- | --- |
| **Procedure** | **Item/condition** | *Mean* | *SD* | *Mean* | *SD* | *p value* |
| Joints/tendons/enthesis/ bursae injection/aspiration | *Use of a sterile cover and/or sterile gel* | 7.77 | 2.95 | 8.15 | 2.75 | ns |
|  | *Aseptic/antiseptic conditions* | 9.32 | 1.68 | 9.48 | 1.04 | ns |
|  | *Aseptic/antiseptic room* | 4.58 | 2.92 | 4.62 | 3.12 | ns |
|  | *Assistance of a second operator* | 5.53 | 2.93 | 5.32 | 3.02 | ns |
|  | *Monitoring of vital signs* | 3.33 | 2.57 | 3.67 | 2.95 | ns |
|  | *Specific technical equipment* | 4.10 | 2.85 | 4.51 | 2.77 | ns |
| Synovial biopsy | *Use of a sterile cover and/or sterile gel* | 9.12 | 2.04 | 9.05 | 2.02 | ns |
|  | *Aseptic/antiseptic conditions* | 9.62 | 1.27 | 9.42 | 1.21 | ns |
|  | *Aseptic/antiseptic room* | 6.30 | 3.27 | 5.90 | 3.43 | ns |
|  | *Assistance of a second operator* | 7.12 | 2.51 | 6.83 | 3.43 | ns |
|  | *Monitoring of vital signs* | 4.77 | 2.56 | 4.54 | 3.43 | ns |
|  | *Specific technical equipment* | 6.12 | 3.18 | 5.83 | 3.31 | ns |
| Spine injections | *Use of a sterile cover and/or sterile gel* | 9.12 | 2.37 | 9.24 | 1.67 | ns |
|  | *Aseptic/antiseptic conditions* | 9.51 | 1.41 | 9.67 | 0.88 | ns |
|  | *Aseptic/antiseptic room* | 6.33 | 3.41 | 5.46 | 3.31 | ns |
|  | *Assistance of a second operator* | 7.45 | 2.93 | 6.41 | 3.29 | 0.015 |
|  | *Monitoring of vital signs* | 6.02 | 3.27 | 4.84 | 3.25 | 0.006 |
|  | *Specific technical equipment* | 6.23 | 3.30 | 4.93 | 3.08 | 0.002 |
| Muscle/nerve biopsy | *Use of a sterile cover and/or sterile gel* | 9.04 | 1.95 | 8.88 | 2.20 | ns |
|  | *Aseptic/antiseptic conditions* | 9.38 | 1.32 | 9.31 | 1.21 | ns |
|  | *Aseptic/antiseptic room* | 5.92 | 3.27 | 5.43 | 3.35 | ns |
|  | *Assistance of a second operator* | 7.92 | 2.54 | 5.51 | 3.22 | <0.001 |
|  | *Monitoring of vital signs* | 5.87 | 3.30 | 3.74 | 3.11 | <0.001 |
|  | *Specific technical equipment* | 6.61 | 3.19 | 4.43 | 3.04 | <0.001 |
| Nerve blockade | *Use of a sterile cover and/or sterile gel* | 8.39 | 2.56 | 8.79 | 2.12 | ns |
|  | *Aseptic/antiseptic conditions* | 9.22 | 1.70 | 9.18 | 1.42 | ns |
|  | *Aseptic/antiseptic room* | 5.73 | 3.36 | 5.23 | 3.25 | ns |
|  | *Assistance of a second operator* | 6,68 | 3.09 | 4.93 | 3.22 | <0.001 |
|  | *Monitoring of vital signs* | 5.59 | 3.24 | 3.86 | 3.14 | <0.001 |
|  | *Specific technical equipment* | 5.78 | 3.31 | 3.96 | 2.90 | <0.001 |
| Soft tissue injections | *Use of a sterile cover and/or sterile gel* | 7.83 | 3.25 | 8.01 | 2.75 | ns |
|  | *Aseptic/antiseptic conditions* | 8.61 | 2.29 | 9.01 | 1.75 | ns |
|  | *Aseptic/antiseptic room* | 3.92 | 3.11 | 3.74 | 3.12 | ns |
|  | *Assistance of a second operator* | 4.5 | 3.10 | 4.44 | 3.30 | ns |
|  | *Monitoring of vital signs* | 3.25 | 2.73 | 2.93 | 2.69 | ns |
|  | *Specific technical equipment* | 3.74 | 3.07 | 3.40 | 2.69 | ns |
| SD, standard deviation; ns, not significant. P values were calculated with the Mann-Whitney U test | | | | | | |

**Online supplementary Figure S1**

**Type of training received by survey respondents according to their specialty**

**
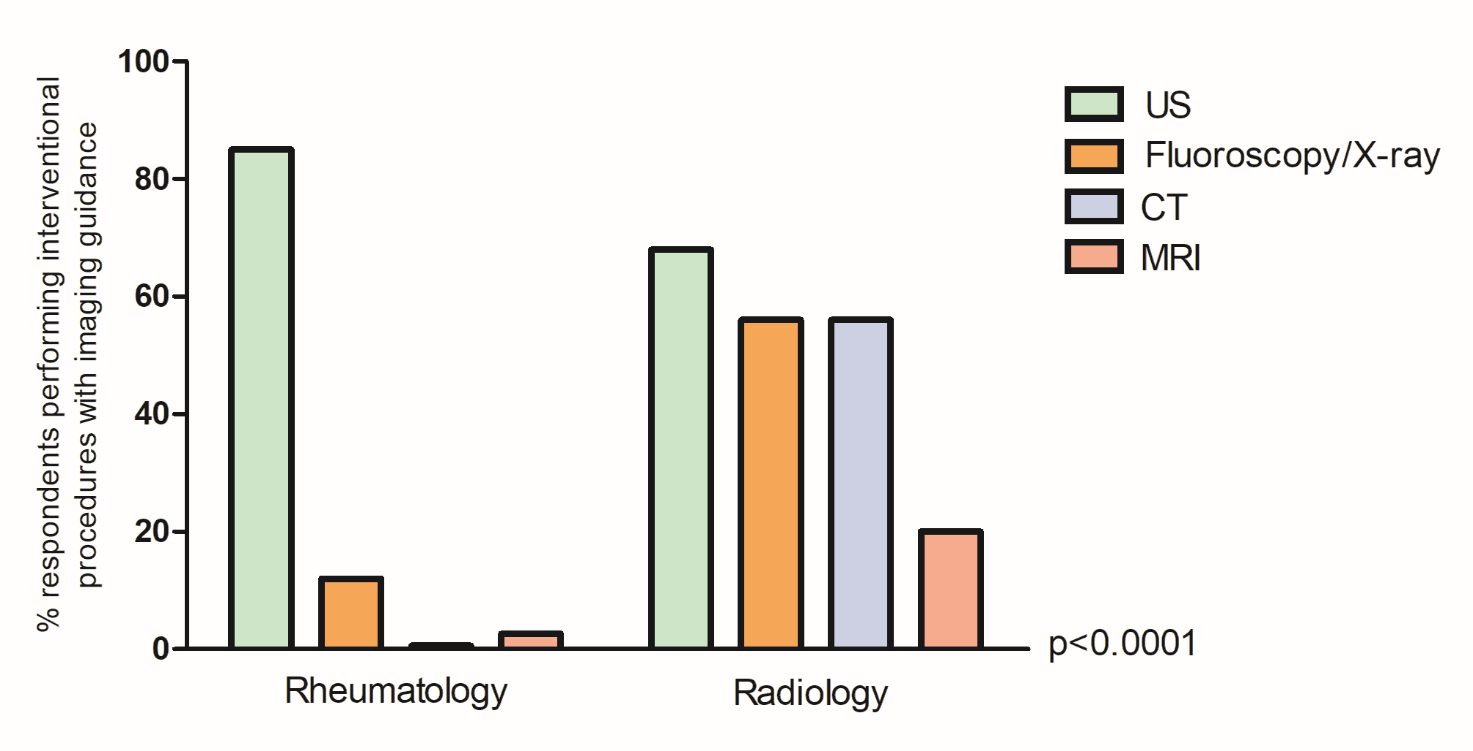
**
